# Implementing two-stage consent pathway in neonatal trials

**DOI:** 10.1136/archdischild-2021-322960

**Published:** 2021-12-23

**Authors:** Eleanor Mitchell, Sam J Oddie, Jon Dorling, Chris Gale, Mark John Johnson, William McGuire, Shalini Ojha

**Affiliations:** 1 Nottingham Clinical Trials Unit, School of Medicine, University of Nottingham, Nottingham, UK; 2 Department of Neonatal Medicine, Bradford Teaching Hospitals NHS Foundation Trust, West Yorkshire, UK; 3 Department of Neonatal Medicine, University Hospital Southampton NHS Foundation Trust, Southampton, UK; 4 Neonatal Medicine, School of Public Health, Faculty of Medicine, Imperial College London, London, UK; 5 NIHR Southampton Biomedical Research Centre, University of Southampton and University Hospital Southampton NHS Foundation Trust, Southampton, UK; 6 Centre for Reviews and Dissemination, University of York, York, UK; 7 Population and Applied Health Sciences, School of Medicine, University of Nottingham, Nottingham, UK; 8 Neonatal Unit, University Hospitals of Derby and Burton NHS Foundation Trust, Derby, UK

**Keywords:** intensive care units, neonatal, neonatology, ethics

## Abstract

Perinatal trials sometimes require rapid recruitment processes to facilitate inclusion of participants when interventions are time-critical. A two-stage consent pathway has been used in some trials and is supported by national guidance. This pathway includes seeking oral assent for participation during the time-critical period followed by informed written consent later. This approach is being used in the fluids exclusively enteral from day one (FEED1) trial where participants need to be randomised within 3 hours of birth. There is some apprehension about approaching parents for participation via the oral assent pathway. The main reasons for this are consistent with previous research: lack of a written record, lack of standardised information and unfamiliarity with the process. Here, we describe how the pathway has been implemented in the FEED1 trial and the steps the trial team have taken to support sites. We provide recommendations for future trials to consider if they are considering implementing a similar pathway. Trial registration number: ISRCTN89654042.

## Abstract (precis)

Short report from a team of UK neonatal triallists highlighting the 2 stage consent process that is being used as part of the ongoing FEED1 trial

**What is already known on this topic?**

- Alternative consent pathways, including oral assent for intervention administration, have been developed in perinatal clinical trials and are supported by national guidance.
- In previous qualitative research, the two-stage consent pathway has shown to be acceptable to parents and healthcare professionals, although some concerns were noted.

**What this study adds?**

- While the two-stage consent pathway is acceptable to healthcare professionals, in the FEED1 trial some recruiters have been hesitant to approach parents to seek oral assent for a variety of reasons.
- This paper provides practical guidance on the implementation of a two-stage consent pathway in a neonatal trial, in order to support recruiters at clinical trial sites.

## Background

Informed consent of participants is a prerequisite for ethical conduct of a clinical trial. In neonatal trials, usually the parents provide consent on behalf of the infant. Studies show most parents believe they ought to make the decision about whether their child participates in research.^1^ Many neonatal trials require time-critical consent, for example, when randomisation is required soon after birth or during acute illness. Parents may be approached antenatally, provided with information about potential participation and then provide consent if the infant’s eligibility is confirmed after birth. This is not always feasible in trials involving preterm infants because preterm birth can be unpredictable. Obtaining valid, informed consent during emergencies or sensitive periods such as when a women has just had an unplanned premature delivery can be difficult and cause stress and emotional distress.^2^

A systematic review that evaluated ethical issues in consenting for clinical trials of preterm or sick neonates (49 studies) concluded that none of the methods available at the time was adequate and alternative approaches were required.^1^ A two-stage consent pathway was developed, in conjunction with NCT (www.nct.org.uk) and Bliss (www.bliss.org.uk), in a clinical trial comparing timing of umbilical cord clamping in very preterm infants and evaluated qualitatively.^3^ This two-stage pathway includes oral assent for randomisation and participation followed by written consent for ongoing use of data. Oral assent was used when there was no opportunity to approach women during the antenatal period, for example, when preterm birth was unpredicted and imminent. This involved obtaining verbal agreement prior to participation and is different to deferred consent, where participants are not approached until after intervention administration as in some emergency medicine trials.^4^ The two-stage process aligns with guidance from the UK Health Research Authority that states that the research consent process should be iterative (https://www.hra.nhs.uk/planning-and-improving-research/best-practice/informing-participants-and-seeking-consent/).

Balancing the complex ethical issues of consent with recruitment can be challenging but is crucial to get right. For clinical trial data to be relevant to the neonatal population of interest, it is important that alternative consent pathways are used. Mother and infant dyads who may not have had the opportunity to consent antenatally are then given the opportunity to participate.^5^

The two-stage pathway developed for the Cord Pilot Trial was acceptable to parents and clinicians.^3^ Although women approached about trial participation reported receiving less information, most felt it was sufficient for decision-making. Clinicians expressed some concerns about how much information to give and the lack of a consent form as a record of the discussion. The two-stage pathway now features in guidance by the Royal College of Obstetricians and Gynaecologists on obtaining consent for perinatal research (https://www.rcog.org.uk/globalassets/documents/guidelines/clinical-governance-advice/clinical-guidance-6a-2016.pdf).

FEED1 is a randomised, multicentre clinical trial comparing feeding full enteral milk feeds from day 1 with gradual milk feeding in infants born at 30^+0^–32^+6^ weeks’ gestational age. Participants are randomised within 3 hours of birth to ensure the allocated feeding strategy is implemented as soon after birth as possible. This period is often stressful and emotional for parents and taking consent can be challenging. A two-stage consent pathway was followed for antenatal consent or oral assent followed by later written consent to make the process less burdensome to families and more feasible for those approaching about consent. The approach was supported by the FEED1 parent and public involvement group and Bliss, the UK’s largest charity for babies born prematurely or sick. This pathway is relatively new in UK neonatal trials and generated some apprehension among those less familiar with the process.

Here, we provide examples of how the two-stage consent pathway is being implemented in the FEED1 trial and suggest recommendations for adopting similar approaches in future studies.

### Implementation and training

The consent pathway is discussed during site training including the background, simulations with role-play, interactive discussions and guidance on the minimal information needed for an oral assent conversation. Two co-investigators who were part of the team that developed the pathway lead this. All training materials are freely available via the trial website (www.feed1.ac.uk).

### Supporting documents

**Figure 1:**
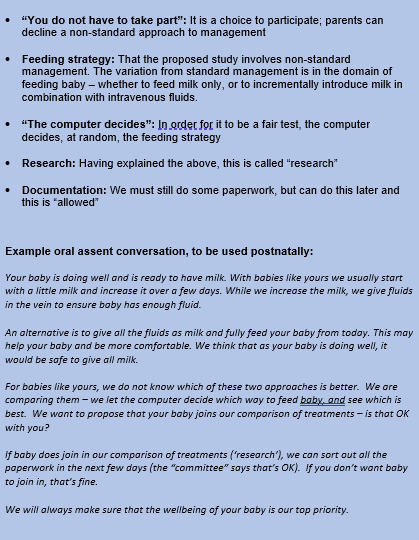
Minimum important information required during an oral assent conversation and example discussion. Created by authors Mitchell and Oddie, permission for reuse granted.

A short document (online supplemental material 1) describing the background with references to supporting national guidance and details of the ethical approval has been prepared and made freely available to sites via the trial website. The minimum important information to include in an oral assent conversation is given in figure 1 with an example discussion.

10.1136/fetalneonatal-2021-322960.supp1 Supplementary data

### Trial webinars

Co-investigators conduct webinars for the sites where assent/consent scenarios are discussed with role-play exercises and exploration of any emerging concerns from the trial recruiters. Webinars are recorded and disseminated to all sites.

### Consent scenarios

Several videos of role-play scenarios of oral assent conversations between clinicians and parents were professionally filmed. The parent ‘actor’ was the parent of a preterm infant. These have been distributed to sites and are available on YouTube and the trial website.

### Oral assent animation for parents

In response to clinicians reporting that the oral assent pathway was impeded by the lack of standardised information and uncertainty about how much information to include in an oral assent conversation, an ethically approved animation for parents, describing the trial, was developed in collaboration with parent partners. It has been distributed to sites with QR code-cards enabling instant access using any internet-enabled device and is available on the trial website.

### Q&A sessions with recruiting sites

The trial team hold monthly meetings with site staff to discuss emerging issues and share experiences. Quarterly clinical discussion forums are also held where, in addition to discussion on other trial issues, the consent process is discussed. Recruiters are given the opportunity to raise barriers and facilitators to approaching parents and conducting oral assent discussions.

## Ongoing approach to trial delivery and conduct

These processes have been developed during the initial phase of recruitment. The trial team work in an iterative manner, adapting the training material and support provided to sites as per the feedback from the recruiters and parent and public partners. Strategies will be evaluated though site feedback has been positive, particularly with respect to the oral assent animation which has been well received by parents.

## Recommendations for future research

Previous research has shown that the two-stage consent pathway is acceptable to parents and clinicians. The FEED1 experience demonstrates that sites require support and resources to implement the pathway successfully. A detailed description of the process should be included in the trial protocol and full ethical approval is mandatory. Early engagement with public and parent partners in developing easily accessible study information resources is crucial. Additionally, ‘buy in’ from the clinical teams that will recruit and deliver the trial is needed early. This should be supported with training and ongoing engagement activities. Free and easily accessible training materials and use of multimedia resources helps disseminate training.

Such resources can be adapted for other clinical trials, particularly perinatal-neonatal research. Evaluation of the strategies implemented in FEED1 is planned when recruitment is complete. Understanding clinicians’ and parents’ views and analysis of recruitment data achieved by the different pathways will improve our understanding of using assent/consent pathways to make clinical trials more accessible to all potential participants.

## Data Availability

Data sharing not applicable as no datasets generated and/or analysed for this study.

## References

[1] Wilman E , Megone C , Oliver S , et al . The ethical issues regarding consent to clinical trials with pre-term or sick neonates: a systematic review (framework synthesis) of the empirical research. Trials 2015;16:502. 10.1186/s13063-015-0957-x 26537492 PMC4634156

[2] Walsh V , Oddie S , McGuire W . Ethical issues in perinatal clinical research. Neonatology 2019;116:52–7. 10.1159/000494934 30947194

[3] Duley L , Dorling J , Ayers S , et al . Improving quality of care and outcome at very preterm birth: the preterm birth research programme, including the cord pilot RCT. Programme Grants Appl Res 2019;7:1–280. 10.3310/pgfar07080 31566938

[4] Woolfall K , Frith L , Dawson A , et al . Fifteen-minute consultation: an evidence-based approach to research without prior consent (deferred consent) in neonatal and paediatric critical care trials. Arch Dis Child Educ Pract Ed 2016;101:49–53. 10.1136/archdischild-2015-309245 26464416 PMC4752644

[5] Owen LS , Davis PG . Parental consent and neonatal delivery room trials: walking an ethical tightrope. Arch Dis Child Fetal Neonatal Ed 2021;106:116–7. 10.1136/archdischild-2020-319355 33436447

