# Supplementary material for: Implementing two-stage consent pathway in neonatal trials
Source: Arch Dis Child Fetal Neonatal Ed. 2021 Dec 23;108(1):79–82. doi: 10.1136/archdischild-2021-322960 (PMC9763226; doi:10.1136/archdischild-2021-322960)
Supplement: Supplementary data [file fetalneonatal-2021-322960supp001.pdf]

SUPPLEMENTARY MATERIAL 1

Oral Assent in the FEED 1 Trial

The FEED1 Trial has an ethically approved two-stage consent process for women. This process was developed in a previous neonatal trial the team led and was found acceptable to women and clinicians. The process is now recommended by the Royal College of Obstetricians and Gynaecologists (RCOG) in their *"Obtaining Valid Consent to Participate in Perinatal Research Where Consent is Time Critical"* guidance. If you want to find out more, there are some references at the bottom of this sheet. We have included a two-stage process since we are keen to recruit women both **antenatally** and **postnatally**. This is to ensure we recruit a broad range of women and babies. The oral assent pathway should be chosen when time is scarce and the provision of full written information to women, at a sensitive and emotional time, might be difficult. Further there is reason to believe that "consent" obtained without sufficient time for discussion and reflection doesn't represent informed consent with its typically understood attributes. The guidance below will help you obtain oral assent (followed by written consent at a later date) for women where this is preferable.

The principles of obtaining oral assent are given below.

- If a woman is not judged able to participate in a full informed consent conversation, but is able to join a conversation (as evidenced by verbal or nonverbal communication) then undertaking the assent process may be applicable.
- Under many or most circumstances postnatal participation without prior discussion will only be via the assent process.
- Under the assent process, the clinician recording assent takes responsibility for randomisation because full explanation and comprehension of the trial is not feasible.
- Assent conversations must be followed by efforts to secure fully informed consent, as per the protocol within 72 hours where appropriate.
- Assent participation may follow a wider or more focused discussion – some conversations may be as inclusive, or nearly as inclusive, as consent conversations

At its most limited, oral assent conversations MUST include:

- **"You do not have to take part":** It is a choice to participate; parents can decline a non-standard approach to management
- **Feeding strategy:** That the proposed study involves non-standard management. The variation from standard management is in the domain of feeding baby – whether to feed milk only, or to incrementally introduce milk in combination with intravenous fluids.
- **"The computer decides":** In order for it to be a fair test, the computer decides, at random, the feeding strategy
- **Research:** Having explained the above, this is called "research"
- **Documentation:** We must still do some paperwork, but can do this later and this is "allowed"

FEED1_Oral_Assent_Guidance_Final_Version _1.0_10_Jan_2020

For guidance on the documentation of the oral assent process please refer to protocol

Turn overleaf for an example oral assent conversation

An example oral assent conversation, to be used postnatally:

Your baby is doing well and is ready to have milk. With babies like yours we usually start with a little milk and increase it over a few days. While we increase the milk, we give fluids in the vein to ensure baby has enough fluid.

An alternative is to give all the fluids as milk and fully feed your baby from today. This may help your baby and be more comfortable. We think that as your baby is doing well, it would be safe to give all milk.

For babies like yours, we do not know which of these two approaches is better. We are comparing them – we let the computer decide which way to feed baby, and see which is best. We want to propose that your baby joins our comparison of treatments – is that OK with you?

If baby does join in our comparison of treatments ('research'), we can sort out all the paperwork in the next few days (the "committee" says that's OK). If you don't want baby to join in, that's fine.

Of course, these words are not intended to be read out verbatim. The intention is to illustrate the kind of content that might be appropriate.

If you have any queries or concerns about the two-stage consent pathway you should contact the FEED1 team in the first instance; feed1@nottingham.ac.uk.

References:

Chhoa, C.Y., Sawyer, A., Ayers, S. *et al.* Clinicians' views and experiences of offering two alternative consent pathways for participation in a preterm intrapartum trial: a qualitative study. *Trials* **18,** 196 (2017) doi:10.1186/s13063-017-1940-5

Sawyer, A., Chhoa, C., Ayers, S. *et al.* Women's views and experiences of two alternative consent pathways for participation in a preterm intrapartum trial: a qualitative study. *Trials* **18,** 422 (2017) doi:10.1186/s13063-017-2149-3

FEED1_Oral_Assent_Guidance_Final_Version _1.0_10_Jan_2020
